# Tracing of streptococcal strains from infant stools across human body sites links site-specific prevalence to adhesins

**DOI:** 10.1128/aem.00196-25

**Published:** 2025-08-11

**Authors:** Ida Ormaasen, Morten Kjos, Melanie Rae Simpson, Torbjørn Øien, Lars Snipen, Knut Rudi

**Affiliations:** 1 Faculty of Chemistry, Biotechnology and Food Science, Norwegian University of Life Sciences, Ås, Norway; 2 Department of Public Health and General Practice, Norwegian University of Science and Technology 8018, Trondheim, Norway; Centers for Disease Control and Prevention, Atlanta, Georgia, USA

**Keywords:** infant gut microbiota, commensal streptococci, strain-level characterization

## Abstract

Streptococci colonize various human body sites, both as commensals and as pathogens. They are early gut colonizers, but we lack strain-level information about the origin and persistence of streptococci in the non-diseased gut. To gain a greater insight into commensal streptococci in the infant gut and their presence in other body habitats, we analyzed samples from mother-infant pairs collected from multiple body sites (stool, oral cavity, vagina, breast milk). We performed whole-metagenome sequencing and isolated streptococci from 100 infant stool samples (collected at 10 days of age). To trace the streptococci at the strain level, we designed selective quantitative PCR (qPCR) primers for seven streptococcal strains, which were then used to screen the corresponding samples from the other body sites of the infants and their mothers. We found that two of the strains investigated, one *Streptococcus parasanguinis* strain and one *Streptococcus vestibularis* strain, were highly prevalent in stool samples obtained from infants and their mothers. Interestingly, the screening revealed that another *S. parasanguinis* strain, closely related to the stool-prevalent strain, displayed a completely different prevalence pattern, being most prevalent in vaginal swabs, breast milk, and oral swabs. A genotypic comparison of these two *S*. *parasanguinis* strains revealed major differences in genes encoding adhesins, suggesting that host surface attachment could be a key factor for the observed differences in body site specificity. Together, our extensive tracing of streptococci across the body sites of 100 infants and their mothers provides strain-level insight into patterns of distribution and identifies streptococcal strains prevalent in stool.

**IMPORTANCE:**

Streptococci thrive on mucosal surfaces and colonize multiple human body sites, including the gut. To understand how streptococci colonize and spread between body site habitats, strain-level information about their prevalence is required; however, such knowledge is currently lacking. In this study, we isolate streptococci and perform metagenome sequencing and quantitative PCR (qPCR) on samples from a large cohort of mother-infant pairs to trace streptococcal strains in different habitats. We demonstrate how different strains prefer specific habitats. For example, we show that two closely related strains, both isolated from stool, are distributed differently across the human body, with one of them prevalent in stool samples and the other more prevalent in other samples. These results emphasize the necessity of strain-level analysis for the identification of true colonizers of a habitat.

## INTRODUCTION

After birth, the infant’s body undergoes an immediate bacterial colonization (1, 2). Among these is *Streptococcus*, a diverse genus containing both beneficial and harmful species that can colonize multiple sites of the human body (3). Shortly after birth, streptococci colonize the infant gut (4–6), with high abundance in the first weeks (7–9). These bacteria remain as members of the gut microbiota throughout life, although their relative abundance significantly decreases within a few months of life (10–12). Studies suggest that streptococci can be transmitted to the infant gut from maternal reservoirs (13–15). Although early-life mechanisms for gut-bacterial sharing have been studied (16), we still lack knowledge about the gut microbiota establishment at the strain level. To identify gut-colonizing streptococci and their potential maternal reservoirs, it is crucial to trace individual strains and further characterize these for phenotypic traits that can explain the colonization.

More than 30 streptococcal species have been isolated from the human body (17). Although some species are pathogenic (18), most species are commensals (17, 19) and may even benefit human health by inhibiting pathogens (20, 21). As facultative anaerobes, streptococci obtain energy by fermenting simple sugars such as glucose and lactose into lactate, both in the presence and absence of oxygen (22). Such versatility is important, since oxygen is depleted in the infant gut in the first days after birth, leading to an anaerobic gut environment and subsequent establishment of obligate anaerobes (4). Some studies indicate that streptococci may have a key role in the adult gut, more specifically in the small intestine. It has been reported that *Streptococcus* is one of the most abundant genera in the ileal microbiota (11, 12, 23), and omics analyses of the ileal streptococci and their metabolic activity imply that these species are involved in the carbohydrate degradation that takes place in the small intestine (19, 24, 25). Considering that streptococci may have an important role in the small intestine microbiota, more knowledge about the gut streptococci is needed.

Streptococci are commonly found in the oral cavity, upper respiratory tract, skin, genitourinary system, and gut (26). The oral cavity is colonized by an abundant streptococcal population (27). Reported members of the oral microbiota include *Streptococcus parasanguinis*, *Streptococcus gordonii, Streptococcus salivarius,* and *Streptococcus vestibularis*. The latter three species are also associated with the gastrointestinal tract (28–30). Other common human streptococcal species include *Streptococcus pneumoniae* in the upper respiratory tract (31) and *Streptococcus agalactiae* in the genitourinary system (32). Moreover, various streptococcal species like *S. parasanguinis, S. salivarius,* and *S. vestibularis* have been found in breast milk (33–35). Different genetic and environmental factors probably influence the body site preference of streptococci, including nutrient availability. Importantly, streptococci produce various adhesins used in host surface attachment (26). These cell surface-associated proteins recognize and bind to receptors found in the different tissues of the human body (36). Therefore, the colonization sites depend on the adhesin targets, in addition to environmental conditions like oxygen and pH levels (26).

Given the widespread presence of the *Streptococcus* genus across the human body, strain-level characterization of the streptococcal populations in different habitats is crucial. Whole-metagenome sequencing has made it more possible to characterize the most abundant bacterial species in a metagenome at the strain level, making it suitable for assessing the prevalence and abundance of specific strains across a collection of samples (37). However, further phenotypic characterization of strain-specific traits, beyond what metagenomics can provide, requires the possession of isolates (38). Furthermore, having isolates enables reconstruction of their genomes, which again allows large-scale tracing of strains by sensitive and low-cost methods such as real-time quantitative PCR (qPCR) screenings (39, 40). This highlights the importance of bacterial cultivation in combination with metagenomic analyses.

**Fig 1:**
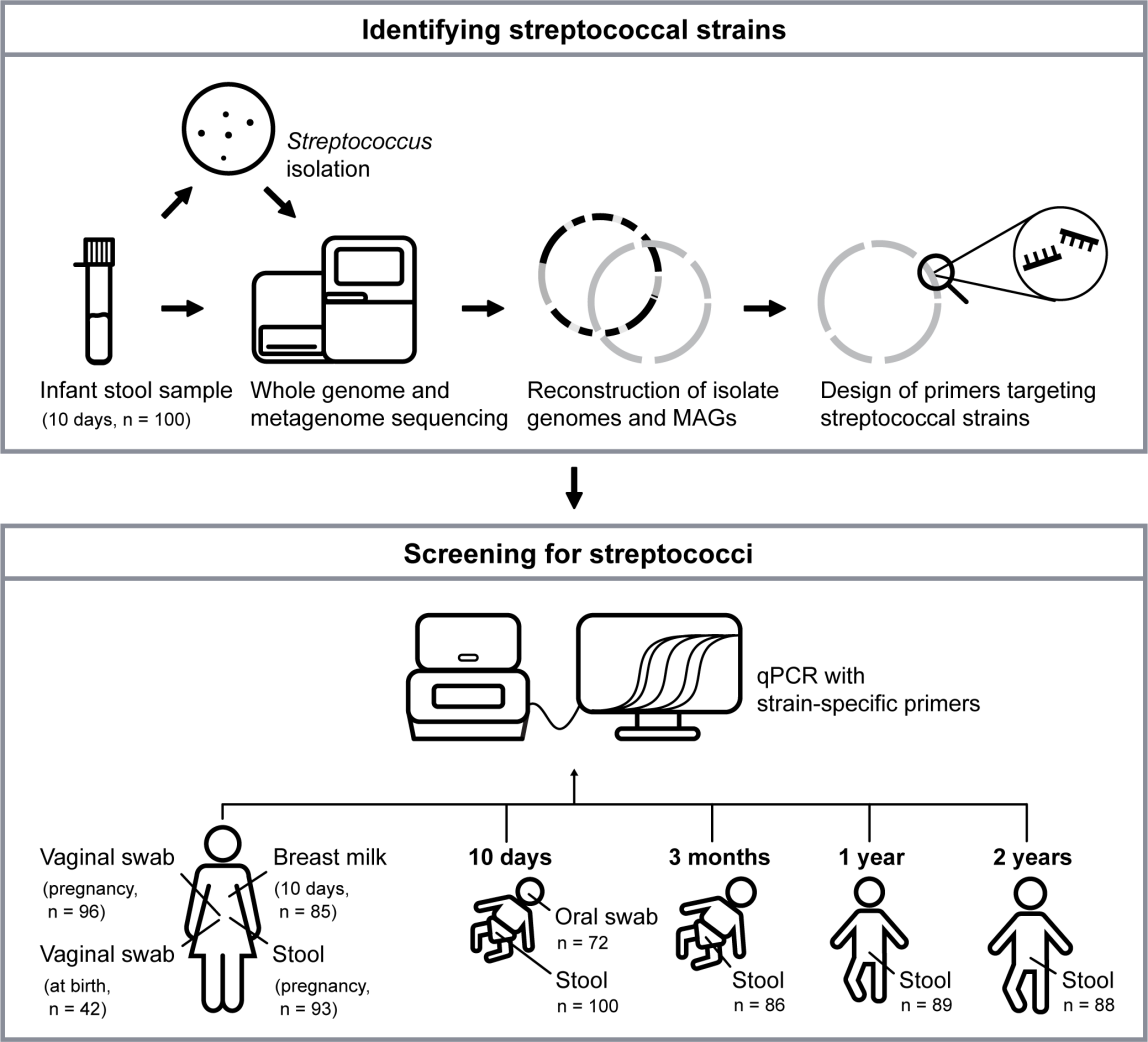
Experimental setup of the streptococcal isolation and screening. Stool samples, collected from 10-day-old infants (*n* = 100), were cultivated on streptococcal-selective agar plates to obtain streptococcal isolates. Whole-metagenome sequencing of the stool samples and whole-genome sequencing of the isolates resulted in the reconstruction of the isolate genomes and streptococcal metagenome-assembled genomes (MAGs). These were used in the design of primers that are specific to each of the streptococcal strains isolated from the stool samples. The designed primers were used in a qPCR screening to enable the detection of the streptococcal strains and subsequent tracing of these across samples from the infants and their mothers (stool, oral swabs, vaginal swabs, breast milk). The different samples had been collected in week 30 of pregnancy and at birth, as well as 10 days, 3 months, 1 year, and 2 years after birth, as shown in the illustration.

Here, we investigated the prevalence of commensal streptococcal isolates across different body sites in 100 mother-infant pairs from the Probiotics in Prevention of Allergy among Children in Trondheim (ProPACT) study in Norway (41). The experimental setup is illustrated in Fig. 1. By performing whole-genome sequencing of streptococcal isolates derived from infant stool samples collected at 10 days of age, combined with whole-metagenomic sequencing of the same samples, we designed primers specific for each strain among the isolates. The strain-specific primers allowed us to perform a comprehensive qPCR-based screen of different samples from the mothers (stool, vagina, breast milk) and their infants (stool, oral cavity), aiming to determine the overall prevalence of the streptococcal strains. Firstly, we present the relative distribution of seven streptococcal strains, of which two are prominent gut-associated strains. Secondly, we suggest that the distinct habitat pattern observed for two closely related strains is partly due to differences in proteins related to host surface attachment.

## RESULTS

### Overall distribution of *Streptococcus* in the different sample types

**Fig 2:**
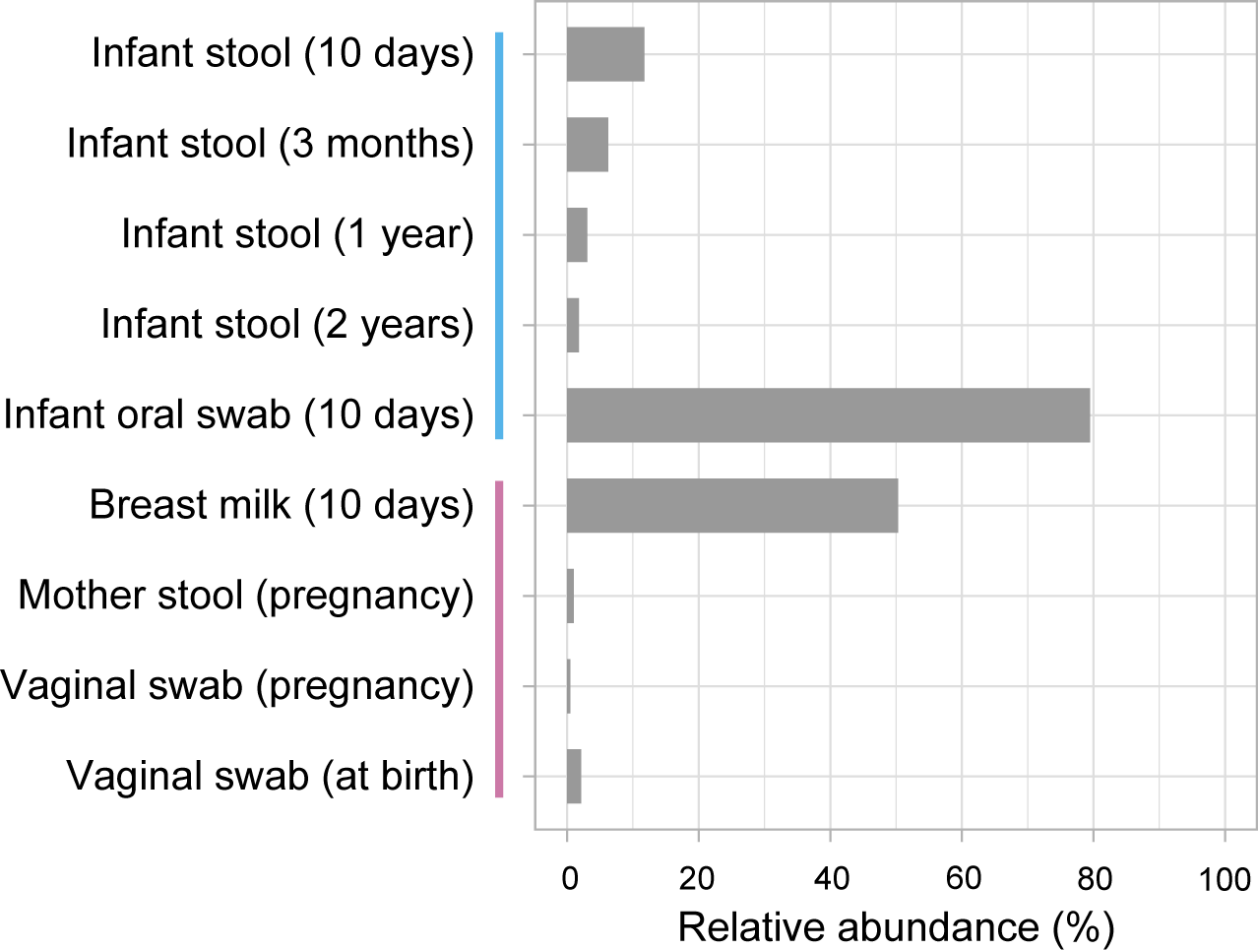
Relative abundance of *Streptococcus*. The figure shows the average relative abundance of *Streptococcus* in the different sample types, determined by 16S rRNA gene analysis. The time points of sample collection are given in parentheses. The vertical lines on the y-axis indicate the samples from the infants (blue) and the mothers (pink).

To investigate the presence of streptococci in the collection of samples, we analyzed previously generated 16S rRNA gene sequencing data (41–44). The analysis identified *Streptococcus* species in all the sample types (Fig. 2). Overall, the highest average relative abundances of streptococci were found in the infant oral cavity (79%) and breast milk (50%). In infant stool, the highest average relative abundance was observed in the samples collected at 10 days of age (12%), followed by those collected at 3 months (6%). In stool samples collected at 1 year and 2 years of age, the average relative abundances had decreased to 3% and 2%, respectively.

### Streptococcal diversity in the infant gut microbiota at 10 days of age

Since the 16S rRNA gene analysis revealed that the highest average streptococcal relative abundance in the infant stool samples was at 10 days of age, we selected these for streptococcal-selective cultivation. Out of the 100 samples, 69 samples displayed growth on the streptococcal-selective agar. After cultivation in liquid medium, a total of 177 bacterial isolates were obtained. These originated from 53 different samples. By Sanger sequencing of the full-length 16S rRNA gene, the most abundant genera among the isolates were identified as enterococci (56%), lactobacilli (18%), and streptococci (9%). Further taxonomic classification of the streptococcal isolates (*n* = 16) was achieved by whole-genome sequencing. This revealed that the isolates belonged to seven species, as defined by GTDB taxonomy: *Streptococcus parasanguinis_D* (*n* = 2), *Streptococcus parasanguinis_I* (*n* = 2), *Streptococcus parasanguinis_F* (*n* = 1), *S*. sp900766505 (*n* = 3), *Streptococcus agalactiae* (*n* = 5), *Streptococcus vaginalis* (*n* = 2), and *Streptococcus vestibularis* (*n* = 1) (Table S1).

Complementary to the cultivation-based approach, we performed whole-metagenome sequencing of the same 100 infant stool samples and subsequent assembly, binning, and genome reconstruction to identify *Streptococcus* spp. The sequencing resulted in 112 metagenome-assembled genomes (MAGs), of which 14 were classified as streptococci (Table S2). Among the streptococcal MAGs, several represented the same species as the isolates (*S. parasanguinis_D* [*n* = 1], *S. agalactiae* [*n* = 1], *S*. sp900766505 [*n* = 1], *S. vaginalis* [*n* = 1], and *S. vestibularis* [*n* = 1]). The remaining streptococcal MAGs were assigned to the following species according to the GTDB taxonomy: *S. parasanguinis_S* (*n* = 1), *S. salivarius* (*n* = 1), *S. infantis_M* (*n* = 1), *S. lactarius* (*n* = 1), *S*. sp001556435 (*n* = 1), *S*. sp000187445 (*n* = 1), and *Streptococcus* spp. (*n* = 3). To investigate the phylogenetic relationship between the streptococcal isolates and MAGs, we determined the estimated evolutionary distances between them. As a reference, we also included complete genomes from the GTDB database that belonged to the same species as the isolates and MAGs. The estimated evolutionary distances, illustrated in the dendrogram in Fig. S1, show that the isolate assemblies cluster according to their assigned species. The same can be seen for most of the MAGs. Furthermore, some of the isolates, including *S. vaginalis* isolate 14, *S. agalactiae* isolate 13, *S. parasanguinis* isolates 8 and 6, and *S. parasanguinis* isolates 5 and 3 (Fig. S1), seem to be identical to a MAG within the same cluster, since the corresponding estimated evolutionary distances are close to zero. Based on the estimated evolutionary distances between the 16 isolates, we concluded that these represent nine different strains, including two *S*. *agalactiae*, three *S*. *parasanguinis* (*S. parasanguinis_D*, *S. parasanguinis_I,* and *S. parasanguinis_F* according to GTDB taxonomy), one *S*. sp900766505, two *S*. *vaginalis,* and one *S*. *vestibularis*. These will be referred to as strains below. To distinguish between the strains belonging to the same species, a number was assigned to their names (Table S1).

**Fig 3:**
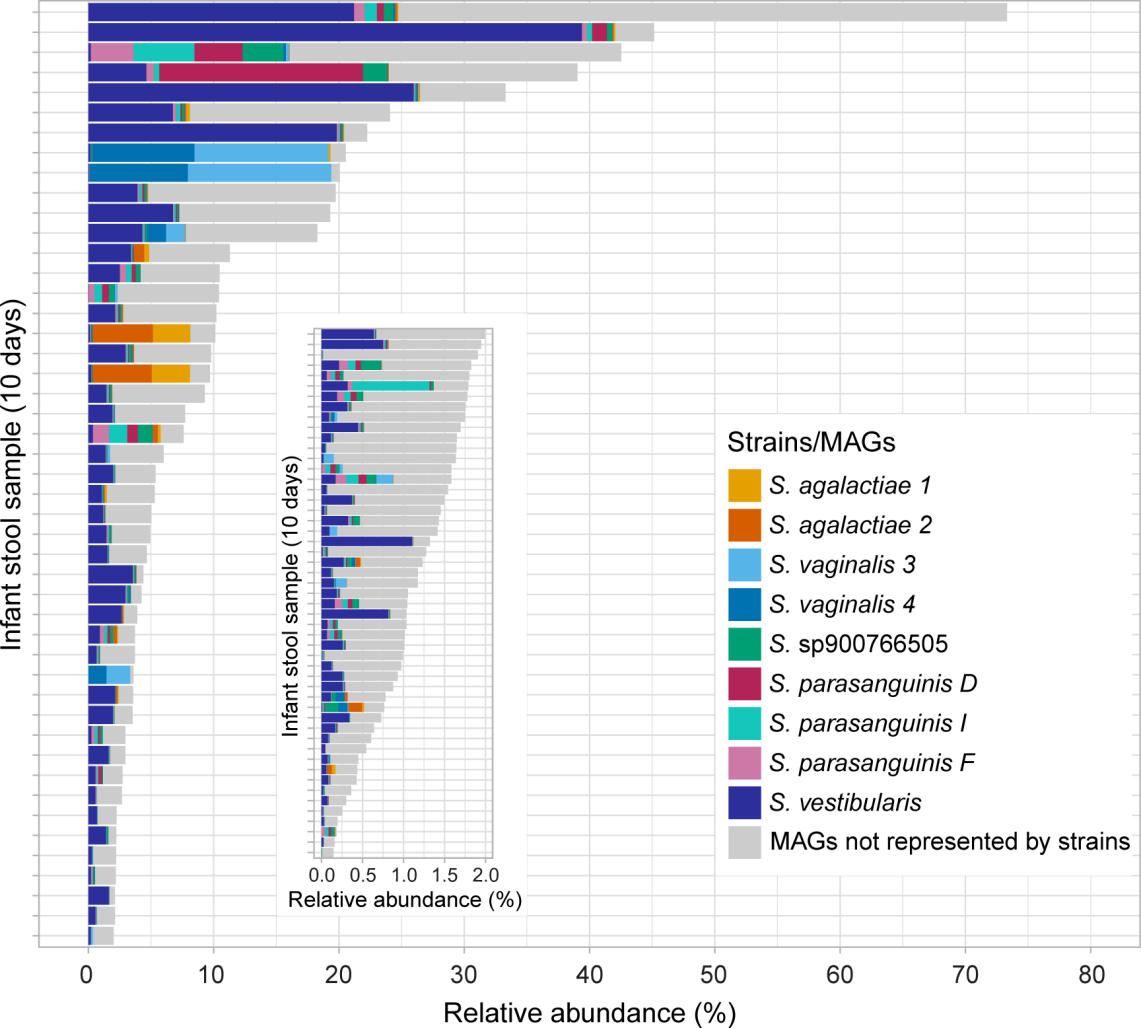
Streptococci in the infant gut at 10 days of age. The relative abundance of streptococci, as determined by whole-metagenome sequencing, is shown. The relative abundances of the nine streptococcal strains in the infant stool samples are indicated in colors. The relative abundances of the streptococcal MAGs with a different taxonomic classification than the nine strains are shown in gray. Note that stool samples with a streptococcal relative abundance of less than 2% are displayed on a different scale.

We investigated the abundance of the nine streptococcal strains and the streptococcal MAGs across the infant stool samples at 10 days of age (see Fig. S2 for total bacterial composition in the infant stool samples). On average, the relative abundance of streptococci in these samples, determined by whole-metagenome sequencing, was 6.3%, and the highest occurring abundance in a sample was 73.3% (Fig. 3). Together, the nine streptococcal strains and the streptococcal MAGs not otherwise represented among the isolate strains contributed to the relative abundance in the samples (Fig. 3). Notably, the *S. vestibularis* strain was extensively more prevalent and abundant than the other strains, contributing to at least 1% of the bacterial composition in 28% of the samples (Fig. 3).

### Streptococcal screening of infant- and mother-derived samples reveals strain-specific prevalence patterns

To study the prevalence of the streptococcal strains across samples collected from the mother-infant pairs, we used strain-specific primers to screen the other samples (Fig. 1). The primers were designed to target genomic regions whose nucleotide sequences were unique to each strain. Systematic testing of the primers showed that seven out of the nine designed primers could successfully be used to specifically identify the targeted strain (we were not able to make strain-specific primers for the strains *S*. sp900766505 and *S. parasanguinis D*). These seven primer pairs were used further in the qPCR-based tracing of strains. The results from the primer design and testing can be found in Table S3.

**Fig 4:**
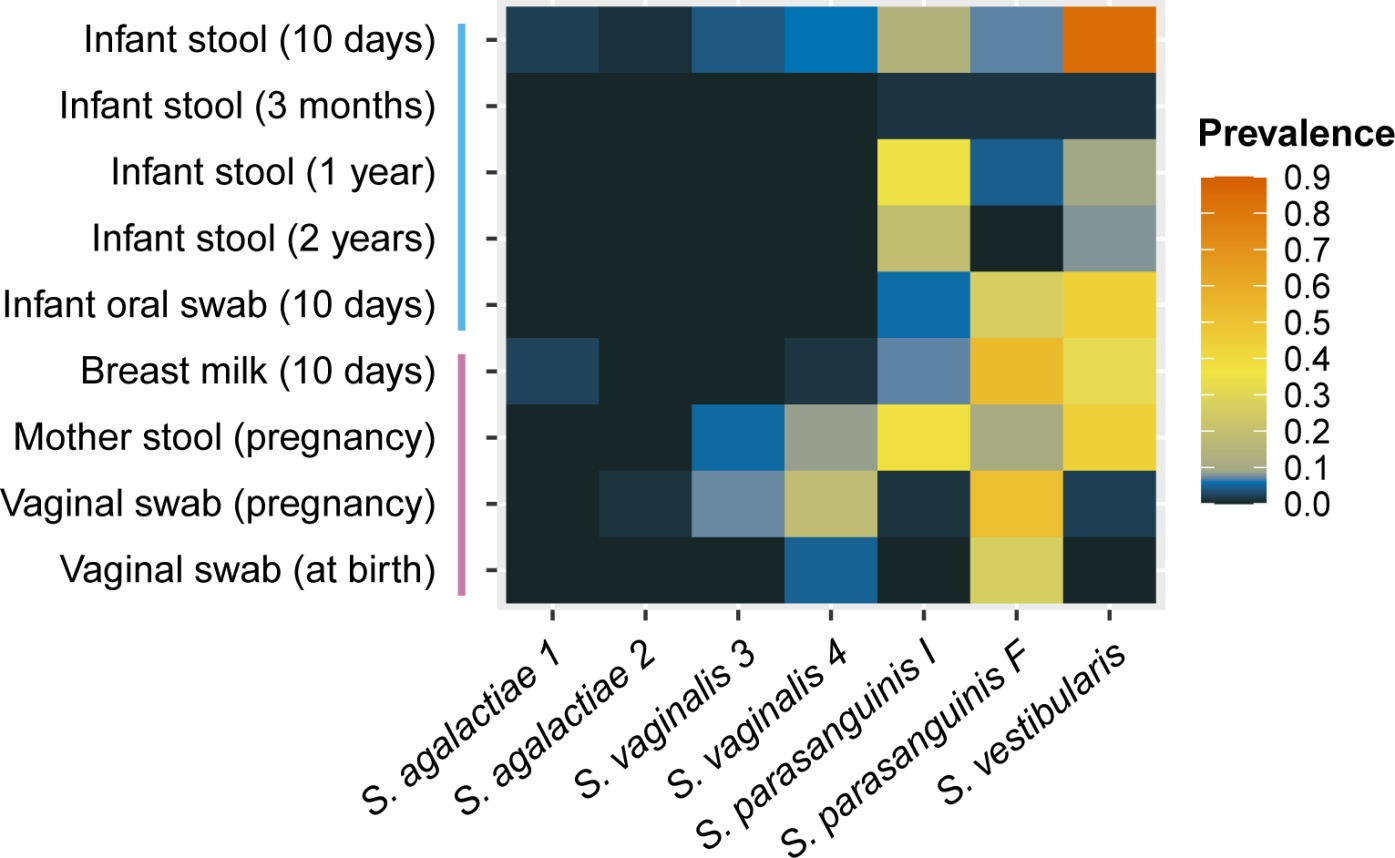
Strain prevalence. Prevalence of the strains in all the samples from maternal and infant body sites at several time points, based on a qPCR screen with strain-specific primers. The time points of sample collection are given in parentheses. The vertical lines on the y-axis indicate the samples from the infants (blue) and the mothers (pink). A detailed overview of the strain detections in each sample is given in Fig. S3.

In line with the metagenome data (Fig. 3), the qPCR screening for the streptococcal strains across the samples revealed that the *S. vestibularis* strain was by far the most prevalent strain in the infant stool samples collected at 10 days of age, being detected in 84% of the samples (Fig. 4). Furthermore, the *S. vestibularis* strain was frequently detected in infant oral samples (44%), breast milk samples (32%), and mothers’ stool samples (44%) (Fig. 4), making it the most prevalent strain across the whole sample collection (27%). The second most prevalent strain was *S. parasanguinis F*, which was found to be present in 19% of the sample collection. This strain was detected across most of the sample types, but it was particularly prevalent in breast milk (53%) and vaginal samples (pregnancy) (51%) (Fig. 4). In contrast, the closely related strain *S. parasanguinis I* displayed a completely different prevalence pattern than *S. parasanguinis F* (Fig. 4). *S. parasanguinis I* was detected mainly in stool samples, both in mothers’ stool and infant stool collected at 10 days, 1 year, and 2 years of age (38%, 14%, 35%, and 19%, respectively). When it comes to the two *S*. *vaginalis* strains, they shared a similar pattern of detection across the samples (Fig. 4). They were primarily detected in mothers’ stool samples, vaginal samples collected during pregnancy, and infant stool samples collected at 10 days of age. The two *S*. *agalactiae* isolates were the least prevalent strains in the screening, with only six samples with detectable levels.

The detection of the same strain from mother and infant samples within mother-infant pairs was common (Fig. S3). There was a statistically significant association between *S. vestibularis* detection in the infant oral cavity and breast milk, with the odds ratio indicating that it was 10.8 times more likely that *S. vestibularis* was detected in breast milk if it was detected in the infant oral cavity as well (Fisher’s test, *P* < 0.005). On the other hand, no statistically significant associations were found between the presence of individual strains in infant stool samples and samples from the other mother or infant body sites. The *P*-values and odds ratios for all tested associations can be found in Table S4.

### Phenotypic and genotypic comparison of the strains

To investigate whether the genetically different strains also exhibited phenotypically distinct traits, their capability to utilize various substrates, including carbohydrates, was tested using the Rapid ID 32 Strep test. Although many of the strains displayed enzymatic activity toward the same substrates, there were differences in substrate utilization among the strains, even between the strains belonging to the same species or closely related species (Fig. S4). Such differences are, for example, seen for the closely related *S. parasanguinis F* and *I* strains, where the latter could utilize additional substrates, including D-ribose, D-mannitol, D-trehalose, D-melibiose, pullulan, resorufin-ß-D-glucopyranoside, and methyl-ß-D-glucopyranoside. Among all the strains, *S. vestibularis* was the only strain capable of utilizing urea.

The strikingly opposite prevalence patterns of the two closely related *S. parasanguinis* strains prompted us to further investigate these genomes in more detail. We compared their genomes to identify dissimilar genes by blasting the genome of *S. parasanguinis I* against *S. parasanguinis F* and vice versa (see Materials and Methods). We anticipated that some genes identified in this process would be the same regardless of which genome was used as the reference or query genome. The comparison resulted in 28 gene hits for *S. parasanguinis I* and 32 for *S. parasanguinis F*, of which 16 of the hits were the same for both strains (Table S5). Notably, 6 of these 16 hits were genes that encode surface proteins and putative adhesins potentially involved in colonization (Table S5). In addition, two adhesin genes were only found in the *S. parasanguinis I* strain, and three other adhesin genes were detected solely in the *S. parasanguinis F* strain. A closer inspection of the sequence alignments and domain organization in these putative adhesins (11 in total) revealed sequence differences and variable numbers of domain repeats (Table S6). For example, we found dissimilarities in surface attachment-related protein domain regions in the genes encoding the GbpC/Spa domain-containing protein, the SspB-related is peptide-forming adhesin, and the CshA/CshB family fibrillar adhesin-related protein, as illustrated in Fig. S5.

## DISCUSSION

Streptococcal species are known to colonize a number of body sites; however, strain-level information is mostly lacking. Here, we performed a large-scale tracing of individual streptococcal strains isolated from stool samples of 10-day-old infants across the body sites of 100 mother-infant pairs. Most prominently, this strain-level mapping revealed streptococcal strains that were prevalent in stool, suggested that the maternal gut microbiota serves as a reservoir for these, and pointed toward genes related to host surface attachment as one of the key drivers for the body site preference of streptococcal strains.

The two strains *S. parasanguinis I* and *S. vestibularis* were highly prevalent in the infant stool samples collected at different time points up to 2 years. Our strain-specific tracing revealed that these strains were also the most prevalent in the stool samples of the mothers, indicating that they are adapted to the human gut environment. In line with these results, Ferretti et al. found that bacterial strains present in mothers’ stool samples were more persistent in the infant gut compared to strains from other sample types (15). Together with our results, this strongly suggests that the maternal gut microbiota serves as an important reservoir for gut-colonizing streptococcal strains in the infant. The ecological niches of streptococci in the gut are not well understood, but it has been suggested that streptococci thrive within the small intestine (11, 19, 24, 25). We specifically looked for the *S. parasanguinis I* and *S. vestibularis* strains in stool samples; however, these species have also been found in effluents from the small intestine (19, 24), indicating that they might be able to occupy niches in the small intestine. It should be noted that the screening performed in this study was limited to certain human body sites, so the body site or habitat preferences of the strains may extend beyond what is reflected in the results.

The dominance of the *S. vestibularis* strain in the 10-day stool samples (Fig. 3) might be the result of an ecological advantage in the initial colonization. Several mechanisms may play a role, but interestingly, we noted that the *S. vestibularis* strain was the only strain capable of utilizing urea, a trait previously reported for the salivarius group (45–47) and linked to enhanced growth (48). Therefore, it is a possibility that utilization of urea in the infant gut by the *S. vestibularis* strain could contribute to a more efficient initial colonization compared to other strains, but this requires further investigation.

The two closely related strains *S. parasanguinis I* and *F* displayed distinct prevalence patterns across the samples from different body sites (Fig. 4). This indicates that, despite their genetic similarity, these strains colonize different habitats. The *S. parasanguinis I* strain occurred mainly in stool samples, while the *S. parasanguinis F* strain was frequently found in the other sample types, including the infant oral samples. The *S. parasanguinis* species is recognized as a common member of the oral microbiota (27), and it has been speculated that translocation from the mouth to the gut can occur (49), resulting in a transient colonization in the gut by this otherwise oral-adapted species (50, 51). Our findings suggest that there is a more nuanced explanation for the presence of *S. parasanguinis* in the gut than previously proposed, possibly involving gut colonization of specific *S. parasanguinis* strains.

The difference in prevalence between body sites seen for the *S. parasanguinis I* and *F* strains was further investigated by substrate utilization tests and by searching for strain-specific variation in their genomes. The two strains displayed differences in substrate utilization capabilities, and this may be a contributing factor to the habitat preferences of the strains. Moreover, the genome comparison also revealed that a major fraction of the genetic variation between the strains was found in genes encoding adhesins or adhesin-associated proteins, such as those orthologous to the GbpC/Spa domain-containing protein, the SspB-related isopeptide-forming adhesin, and the CshA/CshB family fibrillar adhesin-related protein (Table S6). Streptococci are known to produce a great variety of adhesins, and these have been widely studied in relation to the pathogenesis of pathogenic streptococci, mainly in the oral cavity (52). We found genetic variations in regions encoding protein domains involved in surface attachment. An example of this was observed in the GbpC/Spa domain-containing protein, where the number of repeats in the cell surface antigen I/II A repeat domain varied. Orthologous AgI/II proteins are found throughout the *Streptococcus* genus (53). Species-specific variations among these include different numbers of repeats in the alanine-rich repeats (A region) and the proline-rich repeats (P region), as well as dissimilarities in the flanking variable region (V region) (53, 54). Adhesin genes from streptococcal strains within one species have been reported to display high sequence conservation (55, 56). Based on this, it has been suggested that the divergence seen in orthologous AgI/II adhesins is due to niche adaptations evolved in the various species (53, 54). The differences in the prevalence pattern between *S. parasanguinis I* and *S. parasanguinis F* seen in our own results suggest that distinct sets of adhesins used for attachment to host surfaces may also play an important role in the variation in colonization between these two *S*. *parasanguinis* strains. Moreover, these results highlight that more research is needed on adhesins of commensal streptococci, specifically those involved in gut colonization.

Three out of seven strains in the screening were not detected by qPCR in the samples from which they were originally isolated, demonstrating that the strict criteria set in the qPCR analysis affected the strain detection sensitivity. This is partly caused by the chosen study design, in which strict terms were set for the screening analysis to ensure high specificity. Furthermore, the streptococcal-selective cultivation resulted in the revival of only 16 streptococcal isolates, belonging to nine strains. Although the whole-metagenome sequencing identified additional species, the distribution of the streptococcal strains and MAGs in the samples shows that the isolated strains are representative of the streptococcal population in the infant gut 10 days after birth in our study population. Notably, while the same strains were also detected in the infant stool after 1 and 2 years, few detections of these strains were made in the infant stool samples collected at 3 months of age. The 16S rRNA gene sequencing results showed that *Streptococcus* was present in the gut at this time point. The reason for this discrepancy is unknown. A possible explanation may be that the strains found to be prevalent in the stool samples at the other time points are outperformed by other *Streptococcus* species or strains at this stage in the gut microbiota development, before reaching a new bloom within 1 year of age. However, this possible temporal variation needs to be further investigated.

In summary, this comprehensive screening of samples from infant and maternal body sites reveals streptococcal species that are likely to thrive in the human gut environment. Furthermore, closely related species exhibited distinct prevalence patterns across body sites, possibly due to niche adaptations evolved in genes encoding proteins that facilitate host surface attachment. Further research performed on a larger cohort that includes diverse ethnicities, geographical regions, and additional streptococcal isolates is needed to determine phenotypic traits involved in gut microbiota colonization. Particularly, mechanistic studies on adhesins will be essential to determine their contribution to body site specificity among closely related commensal streptococci.

## MATERIALS AND METHODS

### Sample description

The 751 samples in this study were collected from 100 mother-infant pairs participating in the ProPACT study, which represents the normal, healthy population in Norway (41, 57–59). The majority of the infants were born at term and breastfed during the first 3 months post-delivery (41). The infant stool samples were collected 10 days after birth (*n* = 100), 3 months after birth (*n* = 86), 1 year after birth (*n* = 89), and 2 years after birth (*n* = 88), and stored in Cary-Blair transport medium (St. Olavs Hospital, Norway) at −80°C. The infant oral swabs (*n* = 72) were collected 10 days after birth on carbon-impregnated cotton swabs and stored in Stuart Transport medium (60) at −80°C. Stool samples (*n* = 93) from the mothers were collected in week 30 of pregnancy and stored in Cary-Blair transport medium (St. Olavs Hospital, Norway) at −80°C. Vaginal swabs from the mothers were collected at week 30 of pregnancy (*n* = 96) and at birth (*n* = 42) using carbon-impregnated cotton swabs and stored in Stuart Transport medium at −40°C until analysis. Breast milk samples (*n* = 85) were collected 10 days after birth in sterile test containers and stored initially at −20°C and then at −80°C once submitted to the research team. Out of the 100 mother-infant pairs, 20 pairs had a complete set of samples.

### Isolation of *Streptococcus* from infant stool samples collected at 10 days of age

For the selection of streptococci, Streptococcal Selective Agar C.O.B.A. plates (Thermo Scientific Oxoid) were used for cultivation. The 100 infant stool samples were thawed on ice before 50 µL of each sample was spread onto C.O.B.A. plates. The plates were incubated anaerobically, using a GasPak system and AnaeroGen sachets (Oxoid, Thermo Scientific), at 37°C for 2 days. After the incubation, a maximum of five colonies with unique morphologies were picked from each plate and inoculated in 5 mL Todd Hewitt broth (Millipore), followed by an anaerobic incubation in the GasPak system at 37°C overnight. Glycerol stocks containing 20% glycerol were prepared from each culture with visible growth and stored at −80°C. The isolated bacteria were identified through Sanger sequencing of amplicons generated with the cover-all primer pair from Genetic Analysis, which targets the V3-V9 regions of the 16S rRNA gene (61).

### DNA extraction

DNA extraction of infant oral swabs, infant stool samples collected 3 months, 1 year, and 2 years after birth, and mothers’ stool samples was performed prior to this study (41, 43), using the LGC Mag Midi DNA extraction kit (LGC Genomics, UK). DNA extraction of infant stool samples (10 days), vaginal samples, and breast milk samples, as well as the streptococcal isolates and a ZymoBIOMICS Microbial Community Standard (Zymo Research, USA), was performed using the Zymo Research Quick-DNA Fecal/Soil Microbe 96 MagBead kit (Zymo Research, USA). The samples were extracted following the manufacturer’s recommendations, with some exceptions elaborated below. Before the extraction, the sample material was concentrated. Specifically, for the infant stool samples (10 days), 500 µL of each sample was centrifuged at 13,000 rpm for 5 min at 4°C, and the supernatant was then removed. Similarly, 500 µL of each vaginal and breast milk sample was centrifuged at 21,500 × *g* for 30 min at 4°C before the supernatant was removed. Pellets from all these sample types were resuspended in 350 µL lysis buffer (Bashing Bead Buffer) and transferred to FastPrep tubes containing acid-washed glass beads of three sizes; 0.2 g of the <106 µm beads, 0.2 g of the 425–600 µm beads, and 2 of the 2.5–3.5 mm beads (Sigma-Aldrich, Germany). *Escherichia coli* 11775 was used as a positive DNA extraction control, and lysis buffer (Bashing Bead Buffer) was used as a negative DNA extraction control. The samples were treated in a TissueLyser II (Qiagen) for 2 × 2.5 min at 30 Hz and centrifuged at 13,000 × *g* for 1 min. The DNA was eluted in 30 µL elution buffer to ensure adequate DNA concentrations and quantified with the Qubit dsDNA HS assay kit (Thermo Fisher Scientific, USA).

### Library preparations for whole genome and metagenome sequencing

Library preparation of the stool samples (10 days) was performed with the Illumina DNA Prep kit (Illumina, USA), adding 2–250 ng DNA. Extracted DNA from the ZymoBIOMICS Microbial Community Standard and ZymoBIOMICS Microbial Community DNA Standard (Zymo Research, USA) was included. The pooled library was sequenced on NovaSeq6000 (Illumina, USA) at Novogene (UK), and 30 million reads per sample (2 × 150 paired-end reads) were requested. Library preparation of the streptococcal isolates, and the following sequencing on NovaSeq 6000 (Illumina, USA) (2 × 150 paired-end reads) was performed by Novogene (UK).

### Whole genome and metagenome data processing and analysis

The MAGs were constructed as follows: the metagenomic raw reads were filtered and trimmed with BBDuk, which is part of the BBmap software (BBMap version 39.01) (https://sourceforge.net/projects/bbmap/), using the settings found in File S1. The filtered and trimmed reads were mapped to the human genome using Bowtie 2 version 2.4.5 (62) for decontamination of human DNA and assembled with SPAdes version 3.15.5 (63). The binning of assembled contigs was done twice using MaxBin2 version 2.2.7 (64) and MetaBAT2 version 2.15 (65). The MAGs were taxonomically classified using GTDBTk (66) with the release 2.20 version of the GTDB database (67).

Reconstruction of the isolate genomes included filtering, trimming, assembly of reads, and taxonomic classification of assembled contigs, using the same tools and databases as mentioned for the MAGs. To establish the phylogenetic relationship between the isolates, estimated evolutionary distances were calculated using the MASH tool version 2.3 (68). The distances revealed that some of the isolates were phylogenetically identical. Based on this, a new assembly was done where the reads from the identical isolates were combined to increase coverage, resulting in nine strain assemblies.

The phylogenetic relationship between the isolate assemblies, streptococcal MAGs, and genomes in the GTDB database was estimated with MASH distances. From the GTDB database, all genomes from each of the isolate/MAG species were selected. Of these, only the genomes with the highest available assembly quality were used further. The GTDB genomes were clustered based on the MASH distances to find one representative genome for each species. A hierarchical clustering of these representative genomes, isolate assemblies, and MAGs was done based on the MASH distances.

The strain assemblies of *S. parasanguinis I* and *S. parasanguinis F* were compared, aiming to find genetic differences. A blast database was made from the *S. parasanguinis I* assembly using BLAST+ version 2.15.0 (69). Then, the genes in the *S. parasanguinis F* assembly were identified with Prodigal (70) and blasted against the generated database using blastn with default settings. The same process was done the other way around, making a database from *S. parasanguinis F* and blasting the genes from *S. parasanguinis I* against it. The blast tables were filtered, keeping the hit with the best bit score in each case. Looking for dissimilar genes, the hits with an alignment length to query length ratio lower than 0.75 were kept. Among these, there were no ties between the best matches. The hit query sequences were translated to proteins using the translate() function in the microseq R package (https://cran.r-project.org/web/packages/microseq/index.html). For identification, an online blastp search was done against the non-redundant database available at NCBI (71) in July 2024, using default settings. Protein sequence alignments between the matching genes were performed with Clustal Omega version 1.2.4 (72, 73), and the protein domains were identified using InterProScan (74) with the InterPro database (75).

### Primer design and testing

The nine streptococcal strain assemblies were used as template sequences, and the RUCS tool was used for primer generation (76). The specificity of a primer pair was ensured by specifying for each chosen positive genome, a set of negative genomes where potential primers could not produce matches. For each assembly used as positive, the other assemblies, as well as all the MAGs from the infant stool samples, were set as negatives. In cases where a MAG was very similar to an isolate, the MAG was set as positive as well.

The primers were tested *in silico* against all the strain assemblies and MAGs, using Geneious Prime version 2023.1.2 (https://www.geneious.com), to validate their specificity. Similarly, potential binding toward common gut bacterial genomes from the NCBI Reference Sequence Database (Table S7) was investigated using Geneious Prime. Two primer pairs for each strain were chosen based on their specificity and low hairpin melting temperature. These were finally tested with Primer-BLAST (77).

The specificities of the chosen primers were tested further using qPCR, by investigating their ability to amplify DNA from all 16 streptococcal isolates. The annealing temperature of the primers was set to the same as their melting temperature (Tm = 60°C) to keep the occurrence of unspecific binding to a minimum. The reaction mix and amplification program used were the same as described for the streptococcal screening (see below). The best primer pair for each strain was determined by its specificity in the qPCR test run. The primer pair that showed the largest number of cycles between the true signal from the strain in question and the signals from DNA amplification of other strains was selected for further use. The characteristics of each primer can be found in Table S3.

### Screening of streptococcal strains by qPCR

The 751 samples were screened for seven streptococcal strains. The total bacterial load in the samples was determined by amplification of the V3-V4 region of the 16S rRNA gene, using the primer pair PRK341F (5′-CCTACGGGRBGCASCAG-3′) and PRK806R (5′-GGACTACYVGGGTATCTAAT-3′) (78). The master mix for all qPCR runs contained 1× Hot FirePol EvaGreen qPCR Supermix (Solis Biodyne, Estonia) and 0.2 µM of each primer, in a reaction volume of 20 µL. In each run, about 1–4 ng DNA from the isolate in question was included as a positive control, and nuclease-free water was used as a negative control. The amplification was carried out in a CFX96 Touch Real-Time PCR Detection System (Bio-Rad, USA). The initial denaturation step was executed at 95°C for 15 min, followed by 40 cycles of denaturation at 95°C for 30 s and amplification and elongation at 60°C for 60 s (streptococcal strain-specific primers) or amplification at 55°C for 30 s and elongation at 75°C for 45 s (16S rRNA gene primers). A melt curve analysis was included at the end of each run.

The qPCR results were analyzed with LinRegPCR version 2014.x (79) for baseline correction. To distinguish true qPCR signals from unspecific amplification, two criteria were set in the processing of the qPCR results: (i) the melting temperature had to match the melting temperature of the amplicon, and (ii) the Cq value had to be two cycles lower than the negative control. These criteria are rather strict, lowering the streptococcal strain detection sensitivity but ensuring a high specificity.

When a strain was detected in several samples from two different body sites, Fisher’s exact tests were used to test for associations between the samples from the different body sites, i.e., if there is a non-random co-occurrence of the strain in the two body sites. The *P*-values were corrected with a false discovery rate.

### Characterization of substrate utilization capability

The isolates were grown on different substrates in a Rapid ID 32 Strep test (bioMérieux, France). Prior to the test, isolate cultures were grown in Todd-Hewitt broth (Millipore) and incubated anaerobically at 37°C overnight. The cultures were centrifuged to pellet the cells, which were then washed with 1× PBS and centrifuged once more. The cells were resuspended in MilliQ water to a turbidity of approximately McFarland standard 4. Furthermore, the test was carried out according to the manufacturer’s recommendations, and the results were read manually after incubation for 4 h and overnight. For each isolate, parallel tests were performed.

## Data Availability

The whole-genome sequencing data of the 16 streptococcal isolates are available in the NCBI SRA database, with the BioProject ID PRJNA1168056. The 112 MAGs and their corresponding coverage table are available at https://arken.nmbu.no/~larssn/MiDiv/ida/.

## SUPPLEMENTAL MATERIAL

File S1 aem.00196-25-s0001.txt BBDuk settings used for filtering and trimming the raw sequencing reads.

Supplemental material aem.00196-25-s0002.pdf Fig. S1 to S5 and Tables S1, S2, S4, and S7.

Table S3 aem.00196-25-s0003.xlsx Results from primer design.

Table S5 aem.00196-25-s0004.xlsx Results from genomic comparison.

Table S6 aem.00196-25-s0005.pdf Sequence alignments and protein domains of putative adhesins.

ASM does not own the copyrights to Supplemental Material that may be linked to, or accessed through, an article. The authors have granted ASM a non-exclusive, world-wide license to publish the Supplemental Material files. Please contact the corresponding author directly for reuse.
