# Supplementary material for: Tracing of streptococcal strains from infant stools across human body sites links site-specific prevalence to adhesins
Source: Appl Environ Microbiol. 2025 Aug 11;91(9):e00196-25. doi: 10.1128/aem.00196-25 (PMC12442395; doi:10.1128/aem.00196-25)
Supplement: Supplemental material — Fig. S1 to S5 and Tables S1, S2, S4, and S7. [file aem.00196-25-s0002.pdf]

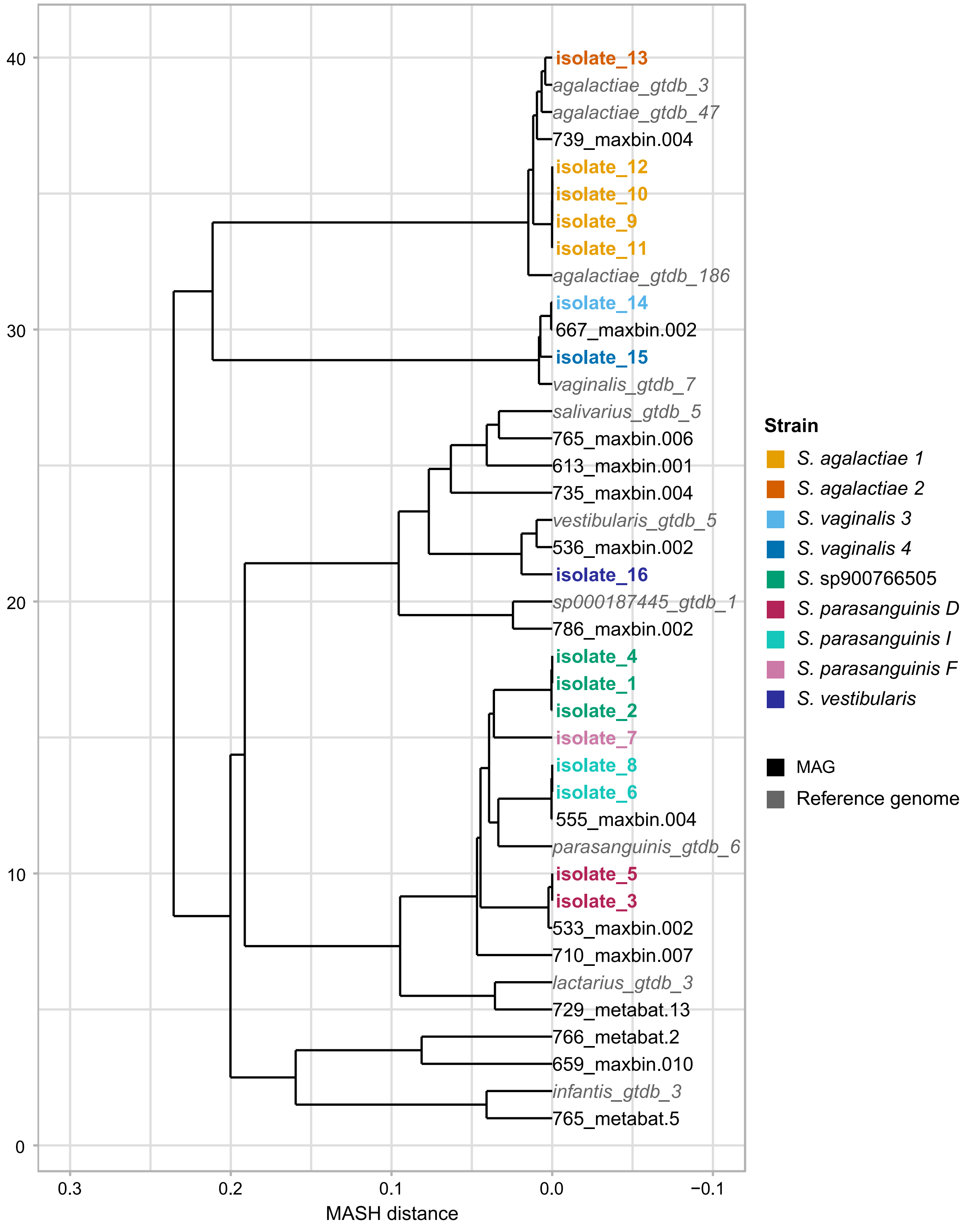

Fig. S1. Phylogeny of streptococcal isolates and MAGs. The mash distances between streptococcal isolates, streptococcal MAGs, and GTDB reference genomes are illustrated in a dendrogram. The isolates are colored according to their strain. The MAGs are shown in black and the GTDB reference genomes are marked in italic and grey.

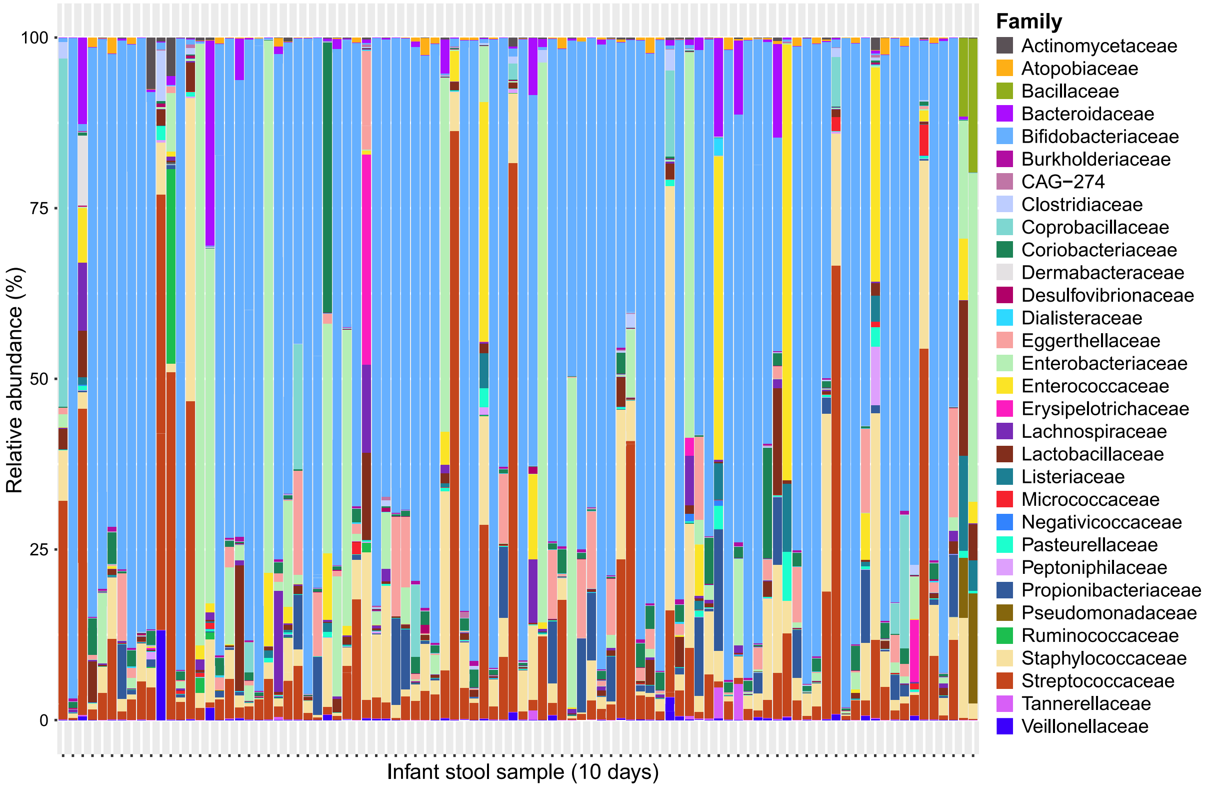

Fig. S2. Bacterial composition in infant stool samples. The figure shows the bacterial composition of the infant stool samples (10 days) at family level, determined through whole metagenome sequencing. Note that about half of the reads in the samples are not accounted for in this figure because they did not get assembled to MAGs.

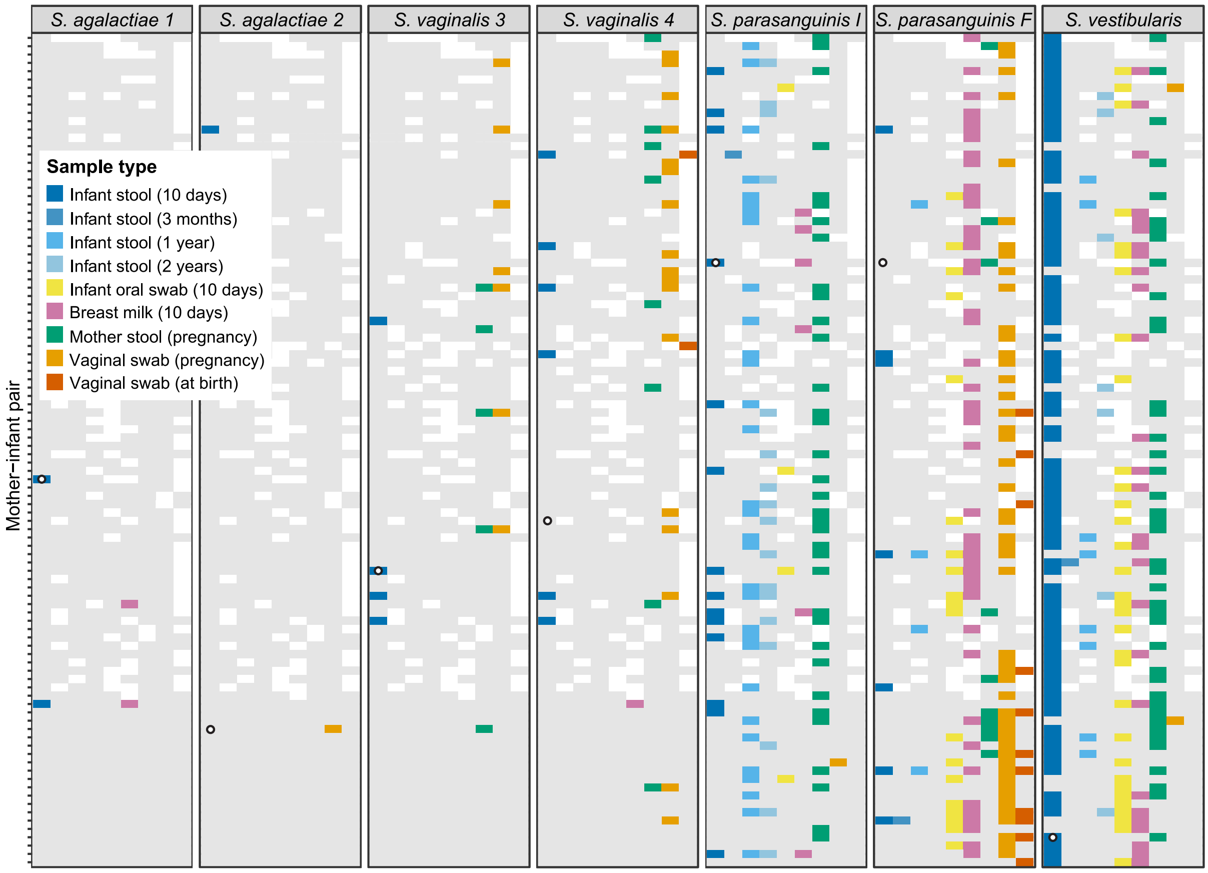

Fig. S3. Streptococcal strain detections across different body sites. The figure shows strain detections in infant stool collected at 10 days, 3 months, 1 year and 2 years of age (dark blue to light blue), infant oral swabs collected at 10 days of age (yellow), breast milk collected 10 days after birth (pink), mother's stool collected during pregnancy (green), vaginal swabs collected during pregnancy (light orange) and vaginal swabs collected at birth (dark orange). Samples where the strains are not detected are marked in grey, and missing samples are marked in white. The white circles indicate from which infant stool sample (10 days) the strains are isolated from.

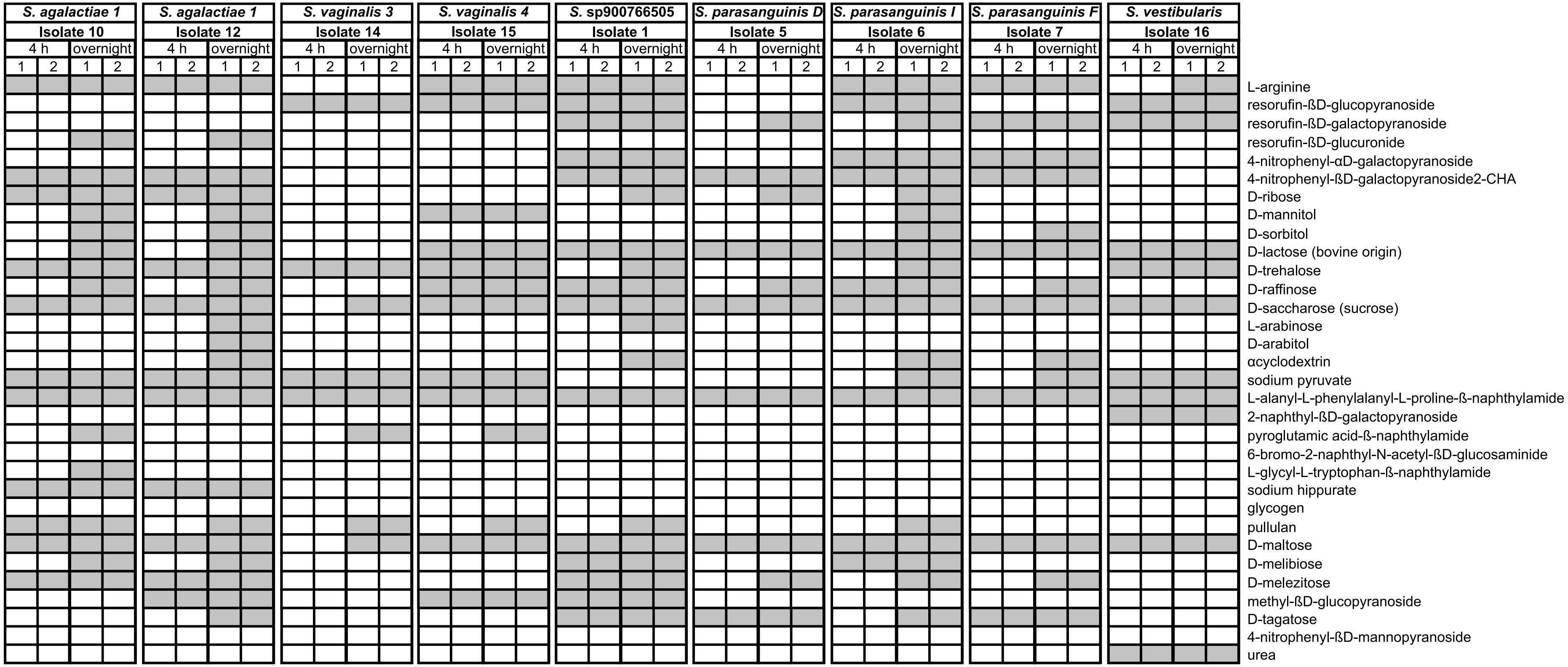

Fig. S4. Substrate utilization. The figure shows the strains capability to utilize different substrates, including a broad variety of carbohydrates. Positive reactions (grey) and negative reactions (white) after 4 hours of incubation and incubation overnight are shown. Parallel tests were performed for each isolate.

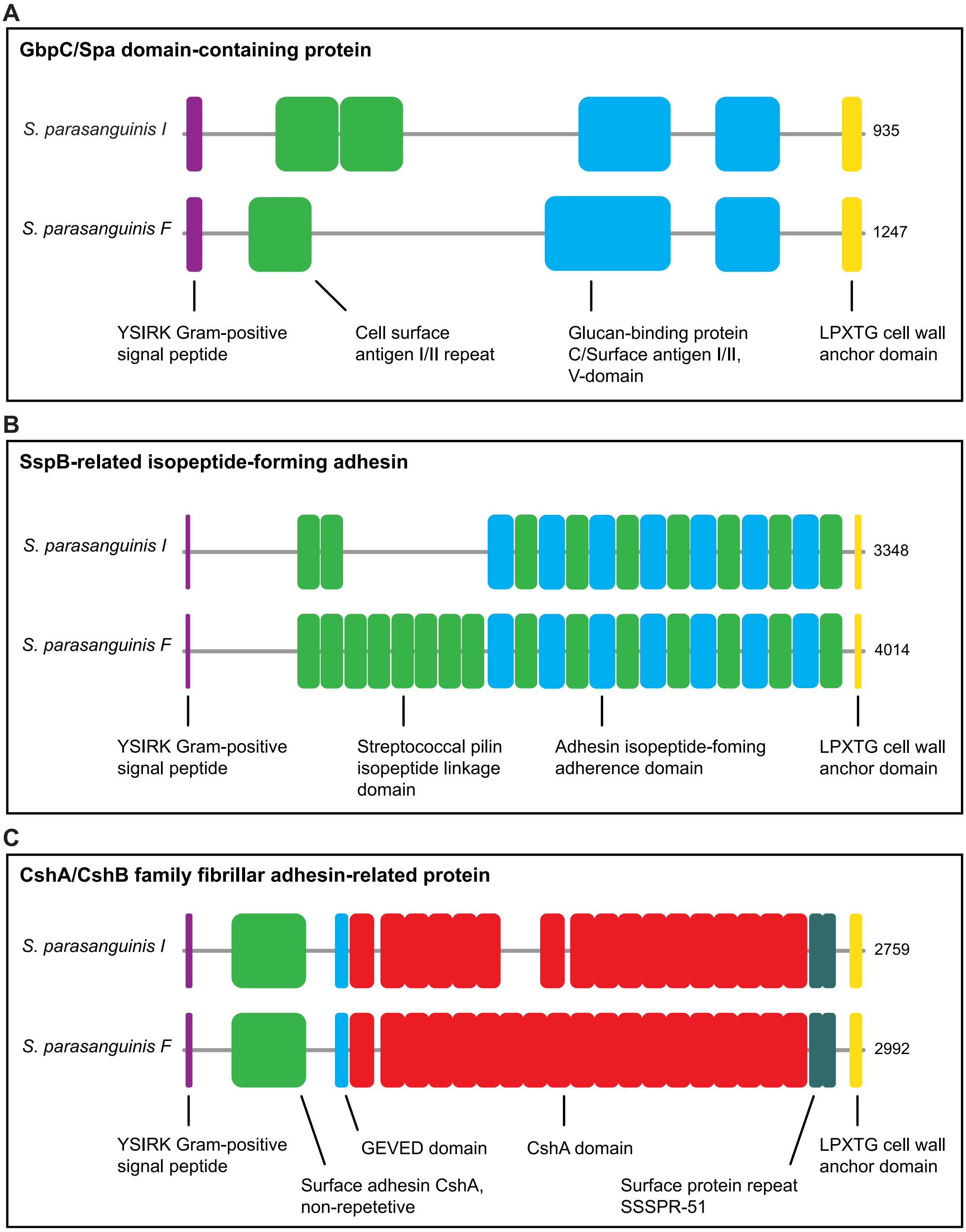

Fig. S5. Illustrations of protein domains in adhesin-related proteins. The figure shows *S. parasanguinis I and F* sequence alignments of protein domains in the GbpC/Spa domain-containing protein (A), the SspB-related isopeptide-forming adhesin (B) and the CshA/CshB family fibrillar adhesin-related protein (C). The colors of the domains correspond to the colors used to mark the same protein domains in the sequence alignments found in Table S6. The protein sequence length is noted at the end of each protein.

Table S1. Taxonomic classification of the streptococcal isolates. The table shows the GTDB database taxonomic classification of the streptococcal isolates.

Isolate	Species	Strain names used in this work
1	*Streptococcus* sp900766505	*Streptococcus* sp900766505
2	*Streptococcus* sp900766505	*Streptococcus* sp900766505
3	*Streptococcus parasanguinis_D*	*Streptococcus parasanguinis D*
4	*Streptococcus* sp900766505	*Streptococcus* sp900766505
5	*Streptococcus parasanguinis_D*	*Streptococcus parasanguinis D*
6	*Streptococcus parasanguinis_I*	*Streptococcus parasanguinis I*
7	*Streptococcus parasanguinis_F*	*Streptococcus parasanguinis F*
8	*Streptococcus parasanguinis_I*	*Streptococcus parasanguinis I*
9	*Streptococcus agalactiae*	*Streptococcus agalactiae 1*
10	*Streptococcus agalactiae*	*Streptococcus agalactiae 1*
11	*Streptococcus agalactiae*	*Streptococcus agalactiae 1*
12	*Streptococcus agalactiae*	*Streptococcus agalactiae 1*
13	*Streptococcus agalactiae*	*Streptococcus agalactiae 2*
14	*Streptococcus vaginalis*	*Streptococcus vaginalis 3*
15	*Streptococcus vaginalis*	*Streptococcus vaginalis 4*
16	*Streptococcus vestibularis*	*Streptococcus vestibularis*

Table S2. Streptococcal MAGs. The table shows the completeness, contamination and taxonomic classification of the streptococcal MAGs. The GTDB database was used for the taxonomic classification.

**MAG id**	**Species**	**Completeness**	**Contamination**
533_maxbin.002	*Streptococcus parasanguinis_D*	91.57	0.22
536_maxbin.002	*Streptococcus vestibularis*	100	0
555_maxbin.004	*Streptococcus parasanguinis_S*	100	0.01
613_maxbin.001	*Streptococcus* sp001556435	100	0.11
659_maxbin.010	*Streptococcus* spp.	87.95	24.13
667_maxbin.002	*Streptococcus vaginalis*	100	0.17
710_maxbin.007	*Streptococcus* sp900766505	82.37	1.86
729_metabat.13	*Streptococcus lactarius*	100	0.07
735_maxbin.004	*Streptococcus* spp.	77.76	22.25
739_maxbin.004	*Streptococcus agalactiae*	100	0.05
765_maxbin.006	*Streptococcus salivarius*	100	0
765_metabat.5	*Streptococcus infantis_M*	96.51	0.72
766_metabat.2	*Streptococcus* spp.	81.46	0.04
786_maxbin.002	*Streptococcus* sp000187445	100	0.06

Table S4. Fisher's test. The table below shows the p-values, adjusted p-values (FDR) and odds ratios for the tested associations between strain detections in samples collected from mothers and infants.

Isolate	Contingency table			p-value	p-value (adjusted, FDR)	Odds ratio
		**breast milk (10 days)**				
S. vestibularis	**oral swab (10 days)**	Detected	Not detected	6.21E-05	0.0025	10.7539
	Detected	18	12			
	Not detected	4	30			
		**mother stool (pregnancy)**				
S. vestibularis	**infant stool (10 days)**	Detected	Not detected	0.1053	0.7702	2.7464
	Detected	18	12			
	Not detected	4	30			
		**breast milk (10 days)**				
S. vestibularis	**infant stool (10 days)**	Detected	Not detected	0.3683	0.9118	2.0706
	Detected	18	12			
	Not detected	4	30			
		**mother stool (pregnancy)**				
S. vestibularis	**oral swab (10 days)**	Detected	Not detected	0.1429	0.7702	0.4565
	Detected	10	19			
	Not detected	21	18			
		**oral swab (10 days)**				
S. vestibularis	**infant stool (10 days)**	Detected	Not detected	1	1	1.1433
	Detected	27	33			
	Not detected	5	7			
		**mother stool (pregnancy)**				
S. vestibularis	**breast milk (10 days)**	Detected	Not detected	0.4666	0.9118	0.6311
	Detected	9	16			
	Not detected	26	29			
		**infant stool (1 year)**				
S. vestibularis	**infant stool (10 days)**	Detected	Not detected	1	1	1.2149
	Detected	7	69			
	Not detected	1	12			
		**infant stool (2 years)**				
S. vestibularis	**infant stool (10 days)**	Detected	Not detected	1	1	1.2507
	Detected	6	67			
	Not detected	1	14			
		**infant stool (2 years)**				
S. vestibularis	**infant stool (1 year)**	Detected	Not detected	1	1	0
	Detected	0	8			
	Not detected	5	65			
		**oral swab (10 days)**				
S. vestibularis	**infant stool (1 year)**	Detected	Not detected	0.69	1	0.5085
	Detected	2	5			
	Not detected	27	34			
		**breast milk (10 days)**				
S. vestibularis	**infant stool (1 year)**	Detected	Not detected	0.4147	0.9118	0.3164
	Detected	1	6			
	Not detected	24	45			
		**mother stool (pregnancy)**				
S. vestibularis	**infant stool (1 year)**	Detected	Not detected	0.449	0.9118	0.4613
	Detected	2	5			
	Not detected	35	40			

S. vestibularis		**oral swab (10 days)**		1	1	1.4154
	**infant stool (2 years)**	Detected	Not detected			
	Detected	2	2			
	Not detected	26	37			
S. vestibularis		**breast milk (10 days)**		0.4207	0.9118	0.3374
	**infant stool (2 years)**	Detected	Not detected			
	Detected	1	6			
	Not detected	22	44			
S. vestibularis		**mother stool (pregnancy)**		0.6937	1	0.5413
	**infant stool (2 years)**	Detected	Not detected			
	Detected	2	5			
	Not detected	32	43			
S. parasanguinis F		**breast milk (10 days)**		0.2574	0.7702	2.2407
	**oral swab (10 days)**	Detected	Not detected			
	Detected	11	6			
	Not detected	21	26			
S. parasanguinis F		**vaginal swab (pregnancy)**		0.8249	1	0.8237
	**breast milk (10 days)**	Detected	Not detected			
	Detected	21	23			
	Not detected	20	18			
S. parasanguinis F		**breast milk (10 days)**		0.2068	0.7702	4.7982
	**infant stool (10 days)**	Detected	Not detected			
	Detected	5	1			
	Not detected	40	39			
S. parasanguinis F		**vaginal swab (pregnancy)**		0.4361	0.9118	2.5332
	**infant stool (10 days)**	Detected	Not detected			
	Detected	5	2			
	Not detected	44	45			
S. parasanguinis F		**vaginal swab (at birth)**		0.1629	0.7702	6.2970
	**infant stool (10 days)**	Detected	Not detected			
	Detected	2	1			
	Not detected	9	30			
S. parasanguinis F		**vaginal swab (pregnancy)**		0.2625	0.7702	3.1597
	**vaginal swab (at birth)**	Detected	Not detected			
	Detected	8	2			
	Not detected	16	13			
S. parasanguinis F		**infant stool (1 year)**		0.02975	0.4073	14.8048
	**infant stool (10 days)**	Detected	Not detected			
	Detected	2	5			
	Not detected	2	80			
S. parasanguinis F		**oral swab (10 days)**		1	1	0.9227
	**infant stool (1 year)**	Detected	Not detected			
	Detected	1	3			
	Not detected	17	47			
S. parasanguinis F		**breast milk (10 days)**		0.1156	0.7702	Inf
	**infant stool (1 year)**	Detected	Not detected			
	Detected	4	0			
	Not detected	35	37			
S. parasanguinis F		**mother stool (pregnancy)**		1	1	0
	**infant stool (1 year)**	Detected	Not detected			
	Detected	0	3			
	Not detected	9	70			
S. parasanguinis F		**vaginal swab (pregnancy)**		1	1	0.9529
	**infant stool (1 year)**	Detected	Not detected			
	Detected	2	2			
	Not detected	42	40			

S. parasanguinis F		**vaginal swab (at birth)**		0.2632	0.7702	Inf
	**infant stool (1 year)**	Detected	Not detected			
	Detected	1	0			
	Not detected	9	28			
S. parasanguinis I		**mother stool (pregnancy)**		0.0434	0.4449	6.6519
	**infant stool (10 days)**	Detected	Not detected			
	Detected	3	10			
	Not detected	3	69			
S. parasanguinis I		**mother stool (pregnancy)**		0.658	1	0.4750
	**breast milk (10 days)**	Detected	Not detected			
	Detected	1	4			
	Not detected	26	49			
S. parasanguinis I		**infant stool (1 year)**		1	1	1.0465
	**infant stool (10 days)**	Detected	Not detected			
	Detected	5	9			
	Not detected	26	49			
S. parasanguinis I		**infant stool (2 years)**		0.2123	0.7702	2.7722
	**infant stool (10 days)**	Detected	Not detected			
	Detected	4	7			
	Not detected	13	64			
S. parasanguinis I		**infant stool (2 years)**		0.258	0.7702	1.9349
	**infant stool (1 year)**	Detected	Not detected			
	Detected	8	21			
	Not detected	8	41			
S. parasanguinis I		**oral swab (10 days)**		1	1	0.7921
	**infant stool (1 year)**	Detected	Not detected			
	Detected	1	19			
	Not detected	3	45			
S. parasanguinis I		**breast milk (10 days)**		1	1	0.9012
	**infant stool (1 year)**	Detected	Not detected			
	Detected	2	25			
	Not detected	4	45			
S. parasanguinis I		**mother stool (pregnancy)**		0.8095	1	1.2004
	**infant stool (1 year)**	Detected	Not detected			
	Detected	11	16			
	Not detected	20	35			
S. parasanguinis I		**oral swab (10 days)**		1	1	0
	**infant stool (2 years)**	Detected	Not detected			
	Detected	0	15			
	Not detected	3	49			
S. parasanguinis I		**breast milk (10 days)**		1	1	1.0569
	**infant stool (2 years)**	Detected	Not detected			
	Detected	1	13			
	Not detected	4	55			
S. parasanguinis I		**mother stool (pregnancy)**		1	1	0.9842
	**infant stool (2 years)**	Detected	Not detected			
	Detected	6	10			
	Not detected	25	41			
S. vaginalis 4		**vaginal swab (pregnancy)**		0.2869	0.7845	2.4700
	**infant stool (10 days)**	Detected	Not detected			
	Detected	2	4			
	Not detected	15	75			
S. vaginalis 4		**vaginal swab (pregnancy)**		0.6309	1	1.5858
	**mother stool (pregnancy)**	Detected	Not detected			
	Detected	2	6			
	Not detected	14	67			

S. vaginalis 3		**vaginal swab (pregnancy)**		0.00287	0.0588	26.8614
	**mother stool (pregnancy)**	Detected	Not detected			
	Detected	3	2			
	Not detected	4	80			

Table S7. Gut bacterial genomes used in primer testing. The gut bacterial genomes listed in the table were downloaded from the NCBI Reference Sequence Database and used for testing the specificity of the designed primers.

NCBI RefSeq assembly / Accession	Species
GCF_000008865.2	*Escherichia coli* O157:H7
GCF_000146185.1	*[Eubacterium] eligens*
GCF_000154385.1	*Faecalibacterium prausnitzii*
GCF_000393015.1	*Enterococcus faecalis*
GCF_000439915.2	*Lactobacillus gasseri*
GCF_002891025.1	*Enterococcus avium*
GCF_006094375.1	*Staphylococcus epidermidis*
GCF_009734005.1	*Enterococcus faecium*
GCF_014131755.1	*Bacteroides thetaiotaomicron*
GCF_018292205.1	*Bacteroides caccae*
GCF_020541885.1	*Bifidobacterium pseudocatenulatum*
GCF_024397795.1	*Lactobacillus intestinalis*
GCF_032486935.1	*Winkia neuii*
NC_000913.3	*Escherichia coli* str. K-12 substr. MG1655
NC_007795.1	*Staphylococcus aureus* subsp. *aureus* NCTC 8325 chromosome
NC_015067.1	*Bifidobacterium longum* subsp. *longum* JCM 1217
NZ_AKCA01000001.1	*Bifidobacterium bifidum* NCIMB 41171 cont1.1
NZ_CP014022.1	*Staphylococcus lugdunensis* strain FDAARGOS_141 chromosome
NZ_CP040780.1	*Lacticaseibacillus rhamnosus* strain 1.0320 chromosome
NZ_CP068170.1	*Thomasclavelia ramosa* strain FDAARGOS_1105 chromosome
